# The Trend of Drug Therapy on Uveitic Macular Edema: A Bibliometric Analysis of the 100 Most Cited Articles

**DOI:** 10.3389/fmed.2022.807319

**Published:** 2022-02-23

**Authors:** Si Chen, Jinfeng Kong, Lei Feng

**Affiliations:** ^1^Eye Center, The Second Affiliated Hospital of Zhejiang University School of Medicine, Hangzhou, China; ^2^Department of Ophthalmology, Jinshan Branch of Shanghai Sixth People's Hospital, Shanghai, China

**Keywords:** macular edema, uveitis, citations, bibliometric analysis, drug treatment

## Abstract

**Background:**

Macular edema is the most common cause of impaired vision due to uveitis. Although various medications are available, not all uveitis patients with macular edema are satisfied with the treatment results. Therefore, solving this gap becomes the utmost concern worldwide. This study attempted to use bibliometric analysis to compare the valuable information in the top 100 highly cited studies in the field of drug therapy for uveitic macular edema (UME) and then determine the research hot spots and trends in this field.

**Methods:**

In this study, the Science Citation Index Expanded (SCIE) of Web of Science (WOS) was used to collect the top 100 most cited studies on UME and analyze the literature from different countries/regions, institutions, and journals. The visualization knowledge maps is generated by VOSviewer and Citespace software.

**Results:**

The top 100 highly cited studies are from 34 countries/regions. The United States has the largest number of publications, followed by the England, Spain and Germany. The top three institutions publishing highly cited literature are all from the England: University of London, University College London, and Moorfields Eye Hospital NHS Foundation Trust. *Ophthalmology* is the most widely published journal with 14 papers. The total number of citations is 1,371, meaning that *Ophthalmology* is the most authoritative journal in the field of UME drug therapy. The top two articles with the most cited times are from the United States, accounting for 36.5% of the total cited times of the top 10 articles. Keywords were divided into three clusters: the corticosteroid administration pathway, biological agents, and clinical trials. Uveitis, cystoid macular edema, efficacy, dexamethasone, and triamcinolone acetonide appeared more frequently in keywords. Researches on local and long-acting drug has gradually becoming the hot spots and trends.

**Conclusion:**

This study concludes that bibliometric analysis can intuitively and quickly obtain the frontiers and hot spots of research in the field of UME drug therapy. Corticosteroid administration, biological agents, and clinical trials are considered the potential focus of future research.

## Introduction

Macular edema is one of the most common complications of uveitis and one of the main causes of blindness in non-infectious uveitis patients (1, 2). According to literature reports, the incidence of macular edema in uveitis patients is about 20–30% or even higher, and more than 1/4 of these patients were with best-corrected visual acuity (BCVA) ≤20/50 during the 2-year follow-up (1, 3).

Nowadays, the pathologic mechanisms and diagnostic classification of UME are relatively clear. In UME, inflammation leads to the destruction of the blood–retinal barrier and increased chorioretinal vascular permeability, resulting in fluid accumulation in the macular area, which is more common in the outer plexus layer (4). Clinically, optical coherence tomography (OCT) can be used to confirm the diagnosis, which can be classified into three types (5, 6): cystoid macular edema (CME), diffuse macular edema (DME), and serous retinal detachment (RD). Among them, DME was the most common, reaching more than half (6). In addition, Iannetti et al. compared the visual impairment between CME and DME patients and found that CME has a greater impact on visual acuity (7).

In contrast, the treatment of UME is a challenging problem due to the diversity of drug therapy. At present, glucocorticoids are still the first-line therapy in clinical practice, and immunosuppressants and biological agents are also being used more and more (8). The Multicenter Uveitis Steroid Treatment Trial (MUST) research group found that intravitreal corticosteroid injections were more effective than periocular injections in the treatment of UME (9). Injectable fluocinolone acetonide implant was used to treat non-infective UME in a clinical study with an average follow-up of 19 months. The implant improved vision and was safe and long-lasting (10, 11). Other studies have shown that for uveitis patients with refractory CME, systemic treatment with interferon alpha-2b can significantly improve CME and vision (12). In recent years, much attention has been paid to the efficacy of biological agents, and there are more and more kinds of biological agents. For example, TNF-α inhibitors are used for refractory cystic macular edema associated with non-infectious uveitis (13). Although there are a variety of drugs used to treat UME in clinical practice, they still cannot meet the needs of all patients. Gaggiano et al. found that patients with juvenile idiopathic arthritis (JIA) related uveitis received a single conventional disease-modifying anti-rheumatic drugs (cDMARDs) does not achieve a good clinical response, especially concurrent UME, which may require a combination of biologic agents, such as interleukin-6 inhibitors, to achieve better therapeutic effect (14). Moreover, there is no sign of active ocular inflammation, macular edema may still persist and even develop into refractory UME, resulting in severe visual impairment. Therefore, UME needs to be further studied in terms of drug therapy.

In recent years, bibliometric analysis has received much attention from researchers. It is a method to study literature publications by using mathematics and statistics and has been widely used in various scientific fields (15). For example, some scholars have used econometric analysis of the literature to determine hot spots and trends in the study of brain inflammasome/coke death (16). Bibliometric analysis can systematically output valuable and reliable information in all relevant literature in a certain field in the form of scientific knowledge maps and tables. Bibliometric analysis is easy to operate, reliable, and reproducible and can prevent the influence of potentially subjective factors. It can help researchers quickly understand the hot spots and trends in related fields and provide new ideas for future research direction planning (4, 16). It is vital for researchers to grasp the research hot spots and emerging development trends in relevant fields in a timely and accurate manner (17). To sum up, bibliometric analysis can precisely meet the needs of researchers.

However, with the rapid development of uveal inflammatory macular edema, research on drug treatment of UME is increasing year by year, but the research hot spot and development trend in this field are still not clear. In this study, bibliometric analysis was applied for the first time in the field of uveal inflammation and macular edema, especially to grasp the hot spots and trends of drug therapy. We used VOSviewer to visualize the top 100 most cited studies in the field of drug therapy for UME in the Web of Science (WOS) database to explore the research direction and potential hot spots of UME in drug therapy.

## Materials and Methods

### Data Sources and Search Strategies

We used the Science Citation Index Expanded (SCIE) of WOS to collect articles. Literature search was performed on October 17, 2021 at Zhejiang University. The search query was framed as follows: (TS = (uveitis) AND TS = (macular edema) AND (TS = (drug therapy) OR TS = (biologicals)OR TS = (Biological Medicine) OR TS = (Biological Drug) OR TS = (Immunosuppressive Agents) OR TS = (Adrenal Cortex Hormones) OR TS = (Glucocorticoids) OR TS = (dexamethasone))). The date range was set from the beginning of the database to October 17, 2021. The search results were arranged by the citation counts in descending order. Then, the 100 most cited documents were selected and sorted in Excel 2019.

### Data Collection

The characteristics of these documents were extracted, including title, keywords, document type, citation number, publication date, country, institutions, journals, and the 2020 impact factor of journals. VOSviewer 1.6.17 (Leiden University, Leiden, the Netherlands) (18) was used to make figures for keyword co-occurrence networks. The circles on the graph represent the keywords, and the diameter of the circles represents the frequency. The larger the diameter of the circle, the higher the frequency of the keyword and the stronger the correlation with the whole. The top 20 keywords with the strongest citation bursts is to import the data of the top 100 highly cited literatures in this field into Citespace 5.8.R3 (19) for analysis. The red bars mean some keywords cited continually; the green bars were keywords cited infrequently.

## Results

### Years and Types of Publication

**Figure 1:**
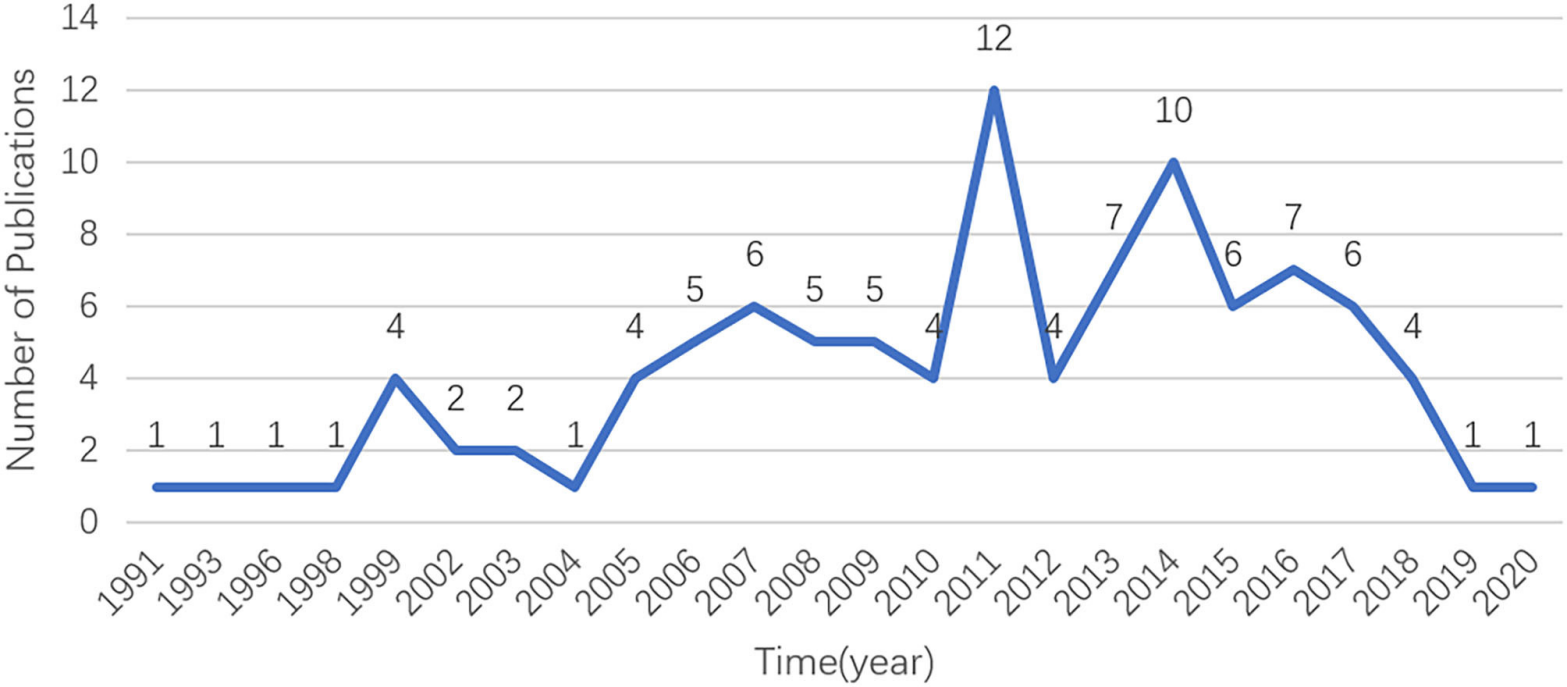
The number of publications in the top 100 most-cited articles per year in the field of UME drug therapy.

A total of 370 documents were acquired from the database by using the search terms given earlier. As shown in Figure 1, the 100 most cited documents were published from 1991 to 2020. The publication time of most of the highly cited documents was from 2005 to 2018. Among them, 12 and 10 papers were published in 2011 and 2014, respectively. The highly cited literature in these two years primarily focused on the treatment of dexamethasone intravitreal implant (DEX implant). This coincided with the time when the DEX implant (Ozurdex; Allergan, Inc., Irvine, CA, USA) was approved by the United States Food and Drug Administration (20). Interestingly, since 2016, the number of highly cited references has decreased significantly, which may be due to the lack of classical literature published in recent years. On the contrary, some studies published earlier did not have more citations due to a longer time.

In terms of document types, Table 1 shows that among the 100 most cited literature studie. Among them, articles account for 60%, reviews account for 31%, proceedings paper accounted for 7%, while book chapters, and editorial materials account for 1% respectively.

**Table 1:** Type of articles of the 100 most-cited articles in the field of UME drug therapy.

**Type of articles**	**Number**
Article	60
Review	31
Article; book chapter	1
Editorial material	1
Article; proceedings paper	7

### Countries/Regions and Institutions Analysis

**Figure 2:**
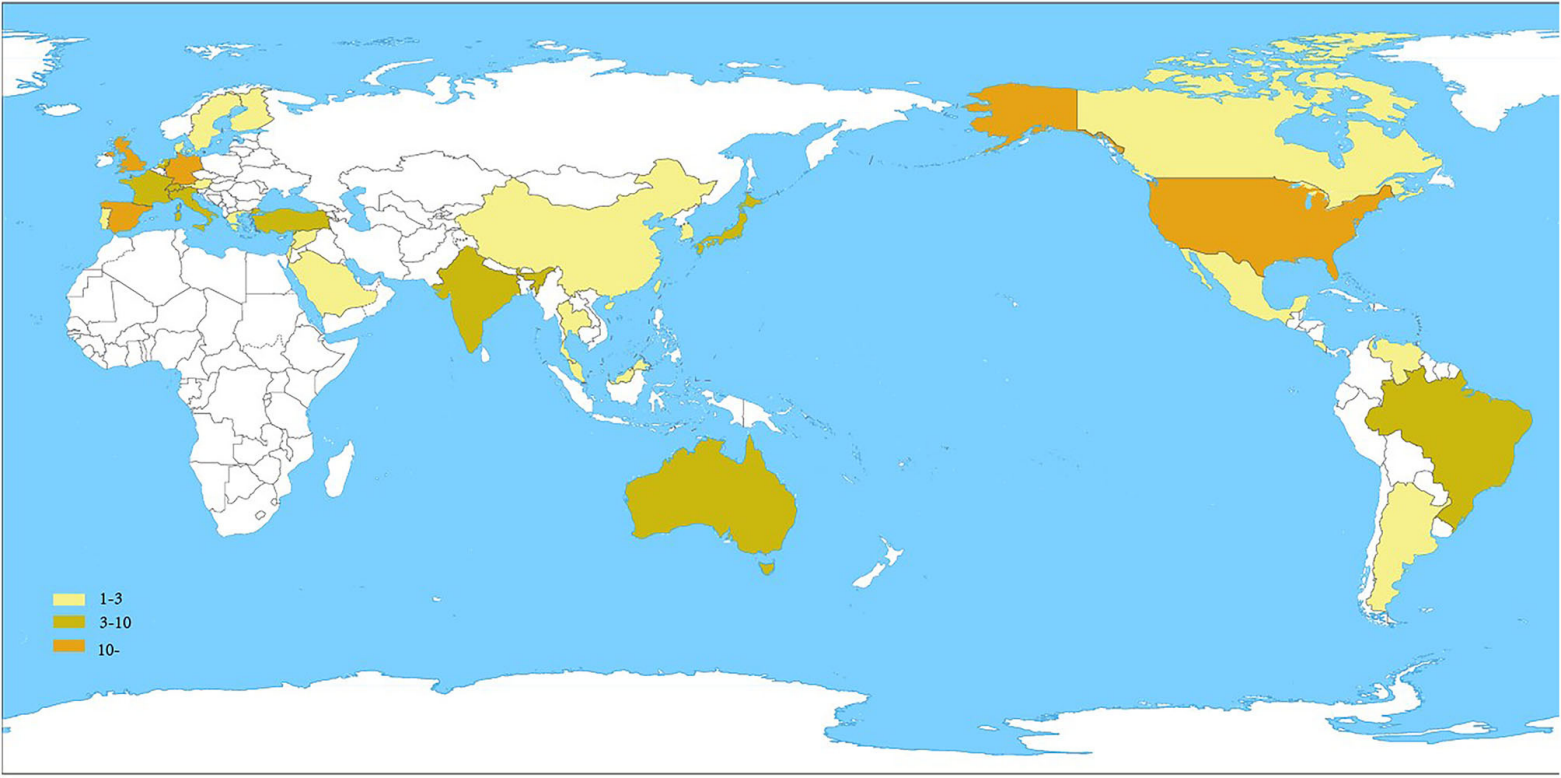
Global spatial distribution of the top 100 highly cited articles published.

Figure 2 summarizes the spatial distribution of 100 highly cited global publications in the field of UME drug therapy. The documents came from 34 countries/regions. Table 2 shows the United States had the largest number of publications, with 46. The England, Spain, and Germany were the next countries/regions to publish more than 10 articles. It can be seen that most of the high-level literature comes from developed countries/regions.

**Table 2:** The top 100 most-cited articles in the field of UME drug therapy by countries/regions and number of publications.

**Country/regions**	**Number**
USA	46
England	17
Spain	16
Germany	11
France	8
Netherlands	8
Italy	7
Switzerland	6
India	4
Japan	4
Australia	3
Brazil	3
Turkey	3
Argentina	2
Greece	2
Malaysia	2
Mexico	2
Peoples R. China	2
Saudi Arabia	2
Thailand	2
Venezuela	2
Austria	1
Canada	1
Costa Rica	1
Denmark	1
Finland	1
Israel	1
Lebanon	1
Portugal	1
Scotland	1
Singapore	1
South Korea	1
Sweden	1
Syria	1

Table 3 shows the top 10 institutions publishing the most cited literature on UME drug therapy and their publications. The top three, notably, are all from the England: University of London (*n* = 12), University College London (*n* = 11), and Moorfields Eye Hospital NHS Foundation Trust (*n* = 11). It is followed by two institutions from Spain: Hospital Clinic Barcelona (*n* = 10) and University of Barcelona (*n* = 10). Also, although the United States has the largest number of highly cited studies, the ranking of institutions in the United States is not the highest.

**Table 3:** Top 10 institutions publishing highly cited literature in the field of UME drug therapy and their publications.

**Rank**	**Institution**	**No. of publications**	**Countries/Regions**
1	University of London	12	England
2	Moorfields Eye Hospital NHS Foundation TrusT	11	England
3	University College London	11	England
4	Hospital Clinic de Barcelona	10	Spain
5	University of Barcelona	10	Spain
6	Abbvie	9	the United States
7	Allergan	9	the United States
8	University of California System	9	the United States
9	Assistance Publique Hopitaux Paris APHP	5	France
10	IDIBAPS	5	Spain

### Journal Analysis

Table 4 lists all journals of these 100 highly cited papers by the number of publications, and 80% of the top 10 journals are from the United States, with the other 20% from the Netherlands and the England. However, the most recent impact factors (IFs) for the top 10 journals ranged from 2.671 to 12.079, with 70% of them in Quartile 1. Of interest, the leading journal was *Ophthalmology* (IF = 12.079) from the United States, with 14% publications and 1,371 citations, indicating that *Ophthalmology* is the authoritative journal in the field of UME pharmacotherapy. The second- and third-ranked journals are *Retinal-The Journal of Retinal* (IF = 4.256) and *American Journal of Ophthalmology* (IF = 5.285). Their publications are 7 and 6, respectively, and their total citations are 305 and 475, respectively. These two journals are also from the United States. These show that the United States has a high degree of influence in this area.

**Table 4:** Journals of the 100 most-cited articles in the field of UME drug therapy.

**Journal**	**No. of articles**	**Citation count**	**IF**	**Quartile in category**	**Countries/regions**
Ophthalmology	14	1,371	12.079	Q1	USA
Retina-The Journal of Retinal and Vitreous Diseases	7	305	4.256	Q1	USA
American Journal of Ophthalmology	6	475	5.285	Q1	USA
Ocular Immunology and Inflammation	6	256	3.07	Q2	Netherlands
Journal of Ocular Pharmacology and Therapeutics	5	227	2.671	Q2	USA
Graefes Archive for Clinical and Experimental Ophthalmology	4	288	3.117	Q2	USA
Investigative Ophthalmology and Visual Science	4	170	4.799	Q1	USA
Archives of Ophthalmology	3	744	4.399	Q1	USA
British Journal of Ophthalmology	3	225	4.638	Q1	England
Current Opinion in Ophthalmology	3	152	3.761	Q1	USA
Eye	3	98	3.775	Q1	England
Ophthalmologica	3	159	3.25	Q2	Switzerland
Documenta Ophthalmologica	2	158	2.379	Q3	Netherlands
European Journal of Ophthalmology	2	56	2.597	Q3	England
Expert Opinion on Pharmacotherapy	2	82	3.889	Q2	England
Ophthalmic Research	2	147	2.892	Q2	Switzerland
Orphanet Journal of Rare Diseases	2	71	4.123	Q2	England
Survey of Ophthalmology	2	145	6.048	Q1	USA
Advanced Drug Delivery Reviews	1	95	15.47	Q1	Netherlands
Advances in Therapy	1	95	3.845	Q3	England
Annals of the New York Academy of Sciences	1	34	5.691	Q1	USA
Arthritis Rheumatology	1	78	10.995	Q1	USA
Biomedicine Pharmacotherapy	1	59	6.529	Q1	France
BMC Ophthalmology	1	32	2.209	Q3	England
Canadian Journal of Ophthalmology Journal Canadian D Ophtalmologie	1	31	1.882	Q4	Canada
Clinical Rheumatology	1	26	2.98	Q3	England
Cochrane Database of Systematic Reviews	1	39	9.266	Q1	England
Current Drug Targets	1	40	3.465	Q2	U Arab Emirates
Drugs	1	34	9.546	Q1	New Zealand
European Journal of Pharmacology	1	27	4.432	Q2	Netherlands
Experimental Eye Research	1	46	3.467	Q2	USA
International Journal of Pharmaceutics	1	41	5.875	Q1	Netherlands
International Ophthalmology Clinics	1	30	0.469 (2001)	Q4	USA
Journal of Cataract and Refractive Surgery	1	40	3.351	Q2	USA
Journal of Drug Targeting	1	32	5.121	Q1	England
Journal of Ophthalmology	1	35	1.909	Q3	USA
Journal of Rheumatology	1	51	4.666	Q2	Canada
Mediators of Inflammation	1	28	4.711	Q2	England
Neurology	1	115	9.91	Q1	USA
Pharmaceutical Science to Improve the Human Condition Prix Galien 2014	1	34	5.691	Q1	USA
Progress in Retinal and Eye Research	1	167	21.198	Q1	England
Proteomics	1	38	3.984	Q2	Germany (FED REP GER)
Rheumatology	1	39	7.58	Q1	England
Scientific World Journal	1	48	1.219 (2013)	Q2	USA
Seminars in Arthritis and Rheumatism	1	53	5.532	Q1	USA
Translational Vision Science Technology	1	31	3.283	Q2	USA

### Cited Literature Analysis

Table 5 lists the top 100 most cited papers in this field, sorted by citation times. Not surprisingly, the top 10 articles of high quality are all in Quartile 1. The total number of citations for the top 10 articles is 1,953. The number of citations for the top two papers, both from the United States, accounted for 36.5% of the top 10 citations. They were, respectively, published by Lowder in 2011 and Kuppermann in 2007, both of which were related to intravitreal injection of dexamethasone in the treatment of UME (19, 20). Also, 5 of the 10 papers are from the United States, 3 from the England, 1 from the Netherlands, and 1 from Spain. We note that Nussenblatt published the earliest of 100 papers in 1991, with 133 citations. He first studied the therapeutic effect of immunosuppressive cyclosporine and steroid hormone in uveitis patients with macular edema (20).

**Table 5:** Top 100 most-cited articles in UME drug therapy studies.

**First author**	**Title**	**Year**	**Journal**	**Quartile in category**	**IF**	**CIT**	**Type**	**Countries/regions**
Lowder, Careen	Dexamethasone Intravitreal Implant for non-infectious Intermediate or Posterior Uveitis	2011	Archives of Ophthalmology	Q1	4.399 (2014)	407	Article	USA
Kuppermann, Baruch D.	Randomized controlled study of an intravitreous dexamethasone drug delivery system in patients with persistent macular edema	2007	Archives of Ophthalmology	Q1	4.399 (2015)	306	Article	USA
Chu, Colin J.	Risk factors and incidence of macular edema after cataract surgery. A database study of 81,984 eyes	2016	Ophthalmology	Q1	12.079	177	Article	England
Kok, H.	Outcome of intravitreal triamcinolone in uveitis	2005	Ophthalmology	Q1	12.079	169	Article	England
de Smet, Marc D.	Understanding uveitis: the impact of research on visual outcomes	2011	Progress in Retinal and Eye Research	Q1	21.198	167	Article	England
de Boer, J.	Visual loss in uveitis of childhood	2003	British Journal of Ophthalmology	Q1	4.638	165	Article	Netherlands
Warwar, R. E.	Cystoid macular edema and anterior uveitis associated with latanoprost use - experience and incidence in a retrospective review of 94 patients	1998	Ophthalmology	Q1	12.079	158	Article	USA
Diaz-Llopis, Manuel	Treatment of refractory uveitis with Adalimumab: a prospective multicenter study of 131 patients	2012	Ophthalmology	Q1	12.079	141	Article	Spain
Nussenblatt, R. B.	Randomized, double-masked study of cyclosporine compared to prednisolone in the treatment of endogenous uveitis	1991	American Journal of Ophthalmology	Q1	5.258	133	Article	USA
Williams, George A.	Dexamethasone posterior-segment drug delivery system in the treatment of macular edema resulting from uveitis or irvine-gass syndrome	2009	American Journal of Ophthalmology	Q1	5.258	130	Article	USA
Yang, Peizeng	Clinical features of Chinese patients with Behcet's disease	2008	Ophthalmology	Q1	12.079	125	Article	Peoples R. China
Jain, Nieraj	Fingolimod-associated macular edema incidence, detection, and management	2012	Neurology	Q1	9.91	115	Review	USA
Erckens, R. J.	Adalimumab successful in sarcoidosis patients with refractory chronic non-infectious uveitis	2012	Graefes Archive for Clinical and Experimental Ophthalmology	Q2	3.117	109	Article	Netherlands
Jonas, Jost B.	Intravitreal triamcinolone acetonide: a change in a paradigm	2006	Ophthalmic Research	Q2	2.892	107	Review	Germany
Kiddee, Weerawat	Intraocular pressure monitoring post intravitreal steroids: a systematic review	2013	Survey of Ophthalmology	Q1	6.048	104	Review	Canada
Moroi, S. E.	Cystoid macular edema associated with latanoprost therapy in a case series of patients with glaucoma and ocular hypertension	1999	Ophthalmology	Q1	12.079	102	Article; Proceedings Paper	USA
Yasukawa, T.	Intraocular sustained drug delivery using implantable polymeric devices	2005	Advanced Drug Delivery Reviews	Q1	15.47	96	Review	Japan
London, Nikolas J. S.	The dexamethasone drug delivery system: indications and evidence	2011	Advances in Therapy	Q3	3.847	95	Review	USA
Zarbin, Marco A.	Ophthalmic evaluations in clinical studies of fingolimod (FTY720) in multiple sclerosis	2013	Ophthalmology	Q1	12.079	89	Article	USA
Diaz-Llopis, Manuel	Adalimumab therapy for refractory uveitis: a pilot study	2008	Journal of Ocular Pharmacology and Therapeutics	Q2	2.671	87	Article	Spain
Adan, Alfredo	Tocilizumab treatment for refractory uveitis-related cystoid macular edema	2013	Graefes Archive for Clinical and Experimental Ophthalmology	Q2	3.117	80	Article	Spain
Wolfensberger, T. J.	The role of carbonic anhydrase inhibitors in the management of macular edema	1999	Documenta Ophthalmologica	Q2	2.379	80	Article; Proceedings Paper	Switzerland
Calvo-Rio, Vanesa	Anti-Interleukin-6 receptor tocilizumab for severe juvenile idiopathic arthritis-associated uveitis refractory to anti-tumor necrosis factor therapy a multicenter study of twenty-five patients	2017	Arthritis and Rheumatology	Q1	10.995	78	Article	Spain
Tomkins-Netzer, Oren	Treatment with repeat dexamethasone implants results in long-term disease control in eyes with non-infectious uveitis	2014	Ophthalmology	Q1	12.079	78	Article	England
Guex-Crosier, Y.	The pathogenesis and clinical presentation of macular edema in inflammatory diseases	1999	Documenta Ophthalmologica	Q2	2.379	78	Article; Proceedings Paper	Switzerland
Zarranz-Ventura, Javier	Multicenter study of intravitreal dexamethasone implant in non-infectious uveitis: indications, outcomes, and reinjection frequency	2014	American Journal of Ophthalmology	Q1	5.258	76	Article	England
Taylor, Simon R. J.	Intraocular methotrexate in the treatment of uveitis and uveitic cystoid macular edema	2009	Ophthalmology	Q1	12.079	76	Article; Proceedings Paper	England
Malcles, Ariane	Safety of intravitreal dexamethasone implant (ozurdex) the safodex study. incidence and risk factors of ocular hypertension	2017	Retina-The Journal of Retinal and Vitreous Diseases	Q1	4.256	71	Article	France
Muselier, Aurore	Efficacy of tocilizumab in two patients with anti-TNF-alpha refractory uveitis	2011	Ocular Immunology and Inflammation	Q2	3.07	71	Article	France
Hsu, Jason	Drug delivery methods for posterior segment disease	2007	Current Opinion in Ophthalmology	Q1	3.761	70	Article	USA
Plskova, Jarka	Interferon-alpha as an effective treatment for non-infectious posterior uveitis and Panuveitis	2007	American Journal of Ophthalmology	Q1	5.258	69	Article	Scotland
Adan, Alfredo	Dexamethasone intravitreal implant for treatment of uveitic persistent cystoid macular edema in vitrectomized patients	2013	Retina-The Journal of Retinal and Vitreous Diseases	Q1	4.256	63	Article	Spain
Taylor, Simon R. J.	New developments in corticosteroid therapy for uveitis	2010	Ophthalmologica	Q2	3.25	63	Article	England
Rathinam, Sivakumar R.	A randomized clinical trial comparing methotrexate and mycophenolate mofetil for non-infectious uveitis	2014	Ophthalmology	Q1	12.079	61	Article	India
Kenawy, Nihal	Abatacept: a potential therapy in refractory cases of juvenile idiopathic arthritis-associated uveitis	2011	Graefes Archive for Clinical and Experimental Ophthalmology	Q2	3.117	61	Article	England
Nayak, Kritika	A review on recent drug delivery systems for posterior segment of eye	2018	Biomedicine and Pharmacotherapy	Q1	6.53	59	Review	India
Mesquida, Marina	Long-term effects of tocilizumab therapy for refractory uveitis-related macular edema	2014	Ophthalmology	Q1	12.079	57	Article	Spain
Edelman, Jeffrey L.	Differentiating intraocular glucocorticoids	2010	Ophthalmologica	Q2	3.25	57	Article	USA
Rothova, Aniki	Inflammatory cystoid macular edema	2007	Current Opinion in Ophthalmology	Q1	3.761	56	Article	Netherlands
Rothova, A.	Medical treatment of cystoid macular edema	2002	Ocular Immunology and Inflammation	Q2	3.07	54	Review	Netherlands
Pato, Esperanza	Systematic review on the effectiveness of immunosuppressants and biological therapies in the treatment of autoimmune posterior uveitis	2011	Seminars in Arthritis and Rheumatism	Q1	5.532	53	Review	Spain
Tappeiner, Christoph	Evidence for tocilizumab as a treatment option in refractory uveitis associated with juvenile idiopathic arthritis	2016	Journal of Rheumatology	Q2	4.666	51	Article	Germany
Zhao, Min	Differential regulations of AQP4 and Kir4.1 by triamcinolone acetonide and dexamethasone in the healthy and inflamed retina	2011	Investigative Ophthalmology and Visual Science	Q1	4.799	50	Article	France
Kato, A	Feasibility of drug delivery to the posterior pole of the rabbit eye with an episderal implant	2004	Investigative Ophthalmology and Visual Science	Q1	4.799	50	Article	Japan
Dutra Medeiros, Marco	Dexamethasone intravitreal implant in vitrectomized versus non-vitrectomized eyes for treatment of patients with persistent diabetic macular edema	2014	Journal of Ocular Pharmacology and Therapeutics	Q2	2.671	49	Article	Spain
Thorne, Jennifer E.	Multifocal choroiditis with panuveitis - incidence of ocular complications and of loss of visual acuity	2006	Ophthalmology	Q1	12.079	49	Article	USA
Kiernan, Daniel F.	The use of intraocular corticosteroids	2009	Expert Opinion on Pharmacotherapy	Q2	3.889	48	Review	USA
Sarao, Valentina	Intravitreal steroids for the treatment of retinal diseases	2014	Scientific World Journal	Q2	1.219 (2013)	47	Review	Italy
Artornsombudh, Pichaporn	Infliximab treatment of patients with birdshot retinochoroidopathy	2013	Ophthalmology	Q1	12.079	47	Article	USA
Barcia, Emilia	Downregulation of endotoxin-induced uveitis by intravitreal injection of polylactic-glycolic acid (PLGA) microspheres loaded with dexamethasone	2009	Experimental Eye Research	Q2	3.467	46	Article	Spain
Okhravi, N.	Cystoid macular edema in uveitis	2003	Ocular Immunology and Inflammation	Q2	3.07	45	Article	England
Cao, Jennifer H.	Dexamethasone intravitreal implant in the treatment of persistent uveitic macular edema in the absence of active inflammation	2014	Ophthalmology	Q1	12.079	42	Article	USA
Whitcup, Scott M.	Pharmacology of corticosteroids for diabetic macular edema	2018	Investigative Ophthalmology and Visual Science	Q1	4.799	41	Review	USA
Rodriguez Villanueva, Javier	Pharmaceutical technology can turn a traditional drug, dexamethasone into a first-line ocular medicine. A global perspective and future trends	2017	International Journal of Pharmaceutics	Q1	5.875	41	Review	Spain
Fardeau, C.	Uveitic macular edema	2016	Eye	Q1	3.775	41	Review	France
Nguyen, Quan Dong	Treating chronic non-infectious posterior segment uveitis: the impact of cumulative damage - Proceedings of an expert panel roundtable discussion	2006	Retina-The Journal of Retinal and Vitreous Diseases	Q1	4.256	41	Article	USA
Schumer, R. A.	Putative side effects of prostaglandin analogs	2002	Survey of Ophthalmology	Q1	6.048	41	Review	USA
Becerra, E. M.	Clinical evidence of intravitreal triamcinolone acetonide in the management of age-related macular degeneration	2011	Current Drug Targets	Q3	3.465	40	Review	Argentina
Tappeiner, C.	Rituximab as a treatment option for refractory endogenous anterior uveitis	2007	Ophthalmic Research	Q2	2.892	40	Article	Germany
Negi, A. K.	Single perioperative triamcinolone injection versus standard postoperative steroid drops after uneventful phacoemulsification surgery - Randomized controlled trial	2006	Journal of Cataract and Refractive Surgery	Q1	3.351	40	Article; Proceedings Paper	England
Ozguler, Yesim	Management of major organ involvement of Behcet's syndrome: a systematic review for update of the EULAR recommendations	2018	Rheumatology	Q1	7.58	39	Review	Turkey
Juthani, Viral V.	Non-steroidal anti-inflammatory drugs versus corticosteroids for controlling inflammation after uncomplicated cataract surgery	2017	Cochrane Database of Systematic Reviews	Q1	9.289	39	Review	USA
Yong, Tao	Intravitreal Triamcinolone	2011	Ophthalmologica	Q2	3.25	39	Review	Germany
Theodossiadis, Panagiotis G.	Intravitreal administration of the anti-TNF monoclonal antibody Infliximab in the rabbit	2009	Graefes Archive for Clinical and Experimental Ophthalmology	Q2	3.117	38	Article	Greece
Deeg, Cornelia A.	Down-regulation of pigment epithelium-derived factor in uveitic lesion associates with focal vascular endothelial growth factor expression and breakdown of the blood-retinal barrier	2007	Proteomics	Q2	3.984	38	Article	Germany
Vegas-Revenga, Nuria	Anti-IL6-receptor tocilizumab in refractory and non-infectious uveitic cystoid macular edema: multicenter study of 25 patients	2019	American Journal of Ophthalmology	Q1	5.258	37	Article	Spain
Denniston, Alastair K.	Heterogeneity of primary outcome measures used in clinical trials of treatments for intermediate, posterior, and panuveitis	2015	Orphanet Journal of Rare Diseases	Q2	4.123	36	Review	England
Minos, Evangelos	Birdshot chorioretinopathy: current knowledge and new concepts in pathophysiology, diagnosis, monitoring and treatment	2016	Orphanet Journal of Rare Diseases	Q2	4.123	35	Review	England
Cabrera, Mariana	Sustained-release corticosteroid options	2014	Journal of Ophthalmology	Q3	1.909	35	Review	USA
Saraiya, Nehali V.	Dexamethasone for ocular inflammation	2011	Expert Opinion on Pharmacotherapy	Q2	3.889	35	Editorial Material	USA
Blumenkranz, Mark S.	Correlation of visual acuity and macular thickness measured by optical coherence tomography in patients with persistent macular edema	2010	Retina-The Journal of Retinal and Vitreous Diseases	Q1	4.256	35	Article	USA
Mesquida, Marina	Twenty-four month follow-up of tocilizumab therapy for refractory uveitis- related macular edema	2018	Retina-The Journal of Retinal and Vitreous Diseases	Q1	4.256	34	Article	Spain
Whitcup, Scott M.	Development of a dexamethasone intravitreal implant for the treatment of non-infectious posterior segment uveitis	2015	Pharmaceutical Science to Improve the Human Condition: Prix Galien 2014	/	/	34	Article; Book Chapter	USA
Gulati, Nishi	Vascular endothelial growth factor inhibition in uveitis: a systematic review	2011	British Journal of Ophthalmology	Q1	4.638	34	Review	USA
Becker, M. D.	Management of sight-threatening uveitis - New therapeutic options	2005	Drugs	Q1	9.546	34	Review	Germany
Chen, Hongming	Recent developments in ocular drug delivery	2015	Journal of Drug Targeting	Q1	5.121	32	Review	USA
Bhagat, Rahul	Comparison of the Release Profile and Pharmacokinetics of Intact and Fragmented Dexamethasone Intravitreal Implants in Rabbit Eyes	2014	Journal of Ocular Pharmacology and Therapeutics	Q2	2.671	32	Article	USA
Tempest-Roe, Shenzhen	Local therapies for inflammatory eye disease in translation: past, present and future	2013	BMC Ophthalmology	Q3	2.209	32	Review	England
Goldstein, Debra A.	Suprachoroidal corticosteroid administration: a novel route for local treatment of non-infectious uveitis	2016	Translational Vision Science and Technology	Q2	3.283	31	Article	USA
Cunningham, Emmett T., Jr.	Practical approach to the use of corticosteroids in patients with uveitis	2010	Canadian Journal of Ophthalmology-Journal Canadien D Ophtalmologie	Q4	1.882	31	Article; Proceedings Paper	USA
Mruthyunjaya, Prithvi	Efficacy of low-release-rate fluocinolone acetonide intravitreal implants to treat experimental uveitis	2006	Archives of Ophthalmology	Q1	4.399 (2016)	31	Article	USA
Schilling, H.	Long-term effect of acetazolamide treatment of patients with uveitic chronic cystoid macular edema is limited by persisting inflammation	2005	Retina-The Journal of Retinal and Vitreous Diseases	Q1	4.256	31	Article	Germany
Holland, G. N.	Immune recovery uveitis	1999	Ocular Immunology and Inflammation	Q2	3.07	31	Article; Proceedings Paper	USA
Khurana, Rahul N.	Efficacy and safety of dexamethasone intravitreal implant for persistent uveitic cystoid macular edema	2015	Retina-The Journal of Retinal and Vitreous Diseases	Q1	4.256	30	Article	USA
Mudumba, Sri	Tolerability and pharmacokinetics of intravitreal sirolimus	2012	Journal of Ocular Pharmacology and Therapeutics	Q2	2.671	30	Article	USA
Samiy, N.	The role of non-steroidal antiinflammatory drugs in ocular inflammation	1996	International Ophthalmology Clinics	Q4	0.469 (2001)	30	Article	USA
Nussenblatt, R.B.	A masked, randomized, dose-response study between cyclosporine-a and cyclosporine-g in the treatment of sight-threatening uveitis of non-infectious origin	1993	American Journal of Ophthalmology	Q1	5.258	30	Article	USA
Bansal, Pooja	Posterior segment drug delivery devices: current and novel therapies in development	2016	Journal of Ocular Pharmacology and Therapeutics	Q2	2.671	29	Review	India
Zierhut, Manfred	Therapy of ocular behcet disease	2014	Ocular Immunology and Inflammation	Q2	3.07	29	Review	Germany
Ghosn, Corine R.	Treatment of experimental anterior and intermediate uveitis by a dexamethasone intravitreal implant	2011	Investigative Ophthalmology and Visual Science	Q1	4.799	29	Article	USA
Hogewind, B. F. T.	Intravitreal triamcinolone for the treatment of refractory macular edema in idiopathic intermediate or posterior uveitis	2008	European Journal of Ophthalmology	Q3	2.597	29	Article	Netherlands
Arcieri, E. S.	The effects of prostaglandin analogs on the blood aqueous barrier and corneal thickness of phakic patients with primary open-angle glaucoma and ocular hypertension	2008	Eye	Q1	3.775	29	Article	Brazil
Pelegrin, L.	Long-term evaluation of dexamethasone intravitreal implant in vitrectomized and non-vitrectomized eyes with macular edema secondary to non-infectious uveitis	2015	Eye	Q1	3.775	28	Article	Spain
Wang, Jillian	Drug delivery implants in the treatment of vitreous inflammation	2013	Mediators of Inflammation	Q2	4.711	28	Review	USA
Dibas, Adnan	Glucocorticoid therapy and ocular hypertension	2016	European Journal of Pharmacology	Q3	2.597	27	Article	USA
Goldberg, I.	A 5-year, randomized, open-label safety study of latanoprost and usual care in patients with open-angle glaucoma or ocular hypertension	2008	European Journal of Ophthalmology	Q3	2.597	27	Article	Australia
Rajesh, Bindu	Safety of 6,000 intravitreal dexamethasone implants	2020	British Journal of Ophthalmology	Q1	4.638	26	Article	Singapore
Lopalco, Giuseppe	IL-6 blockade in the management of non-infectious uveitis	2017	Clinical Rheumatology	Q3	2.98	26	Review	Italy
Adeniran, Janelle M. Fassbender	Common and rare ocular side-effects of the dexamethasone implant	2017	Ocular Immunology and Inflammation	Q2	3.07	26	Review	USA
Pearce, William	Advances in drug delivery to the posterior segment	2015	Current Opinion in Ophthalmology	Q1	3.761	26	Review	USA

### Keyword Analysis

Table 6 lists the top 20 keywords with the highest frequency and strongest correlation strength in the 100 studies. Among them, the top 10 keywords are uveitis (*n* = 37), cystoid macular edema (*n* = 33), macular edema (*n* = 24), therapy (*n* = 16), efficacy (*n* = 15), dexamethasone (*n* = 12), posterior uveitis (*n* = 12), retinal vein occlusion (*n* = 12), diabetic macular edema (*n* = 11), and triamcinolone acetonide (*n* = 11). In general, the higher the occurrence frequency of keywords, the higher the corresponding overall correlation strength. From the perspective of these high-frequency keywords, the research hot spot in this field is mainly reflected in the effect of UME drug therapy, and the research on steroid hormones is relatively active.

**Table 6:** The top 20 keywords with the highest occurrence and strongest overall association strength in articles related to UME drug therapy research.

**Keywords**	**Occurrences**	**Total link strength**
Uveitis	37	216
Cystoid macular edema	33	189
Macular edema	24	148
Therapy	16	75
Efficacy	15	83
Dexamethasone	12	84
Retinal vein occlusion	12	97
Posterior uveitis	12	78
Diabetic macular edema	11	79
Triamcinolone acetonide	11	64
Triamcinolone	10	76
Endothelial growth factor	10	73
Intravitreal triamcinolone acetonide	10	68
Corticosteroids	10	67
Disease	10	50
Standardization	9	52
Dexamethasone intravitreal implant	9	51
Pars plana vitrectomy	8	66
Intermediate	8	50
Fluocinolone acetonide implant	8	45

**Figure 3:**
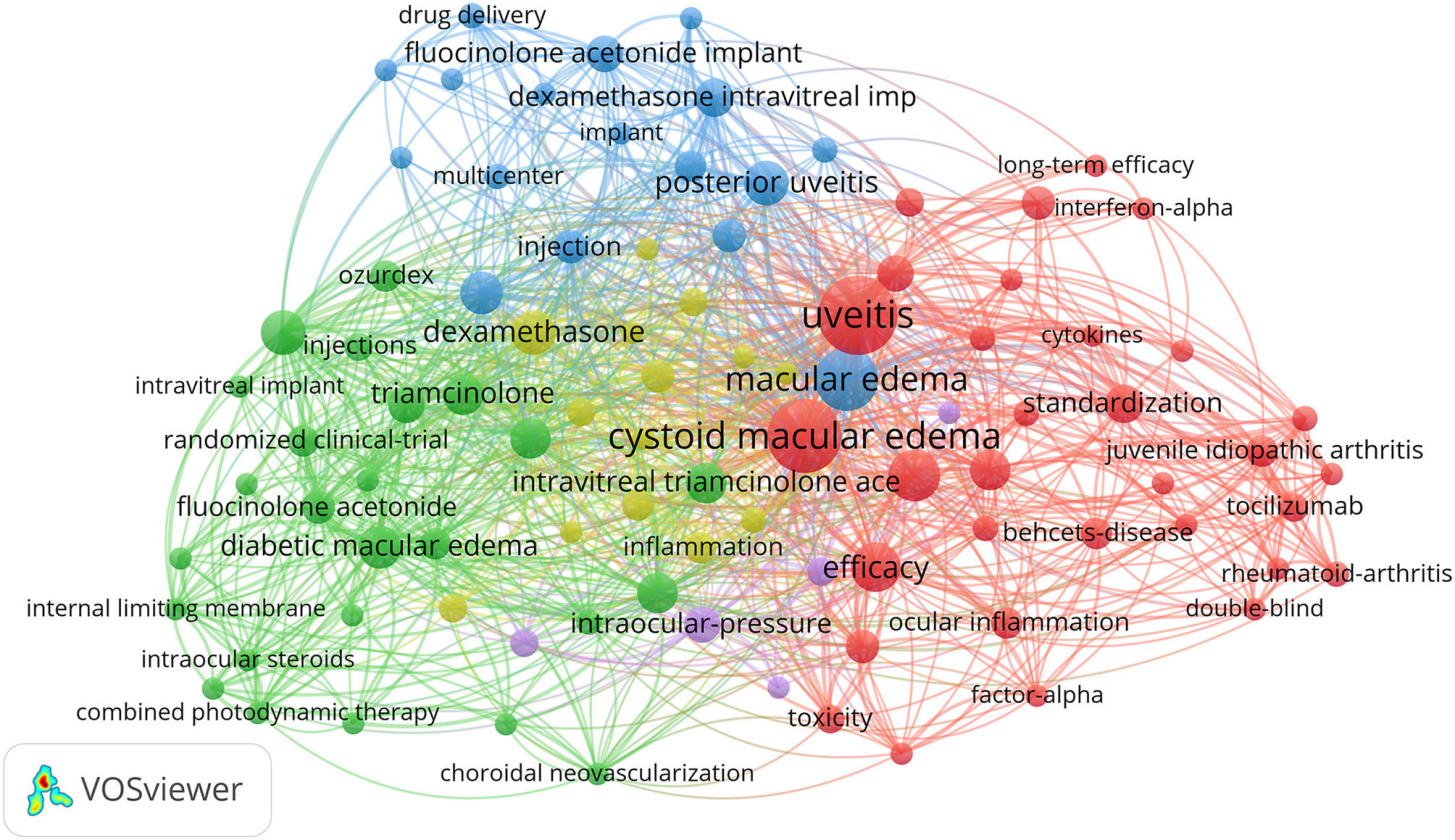
VOSviewer visualization of keywords from the top 100 highly cited articles in the field of UME drug therapy.

VOSviewer software was used to make a keyword co-occurrence network diagram, which can be very intuitive and quickly obtain the frontier and hot spots of a certain field of research. As shown in Figure 3, in the top 100 studies, a total of 93 keywords appeared more than three times. Obviously, these keywords are mainly divided into three clusters and are represented in different colors. The three clusters represent (1) the corticosteroid administration pathway in green, such as intravitreal implant, intravitreal triamcinolone acetonide, corticosteroids, and intraocular steroids; (2) biological agents in red, such as therapy, efficacy, infliximab, and interleukin-6; and (3) clinical trials in blue, such as trial, drug delivery, and multicenter. Also, there are two clusters of lower frequencies of purple and yellow, ocular hypertension, acetazolamide, and open-angle glaucoma. Recent hot spots and trends are reflected in these three aspects.

**Figure 4:**
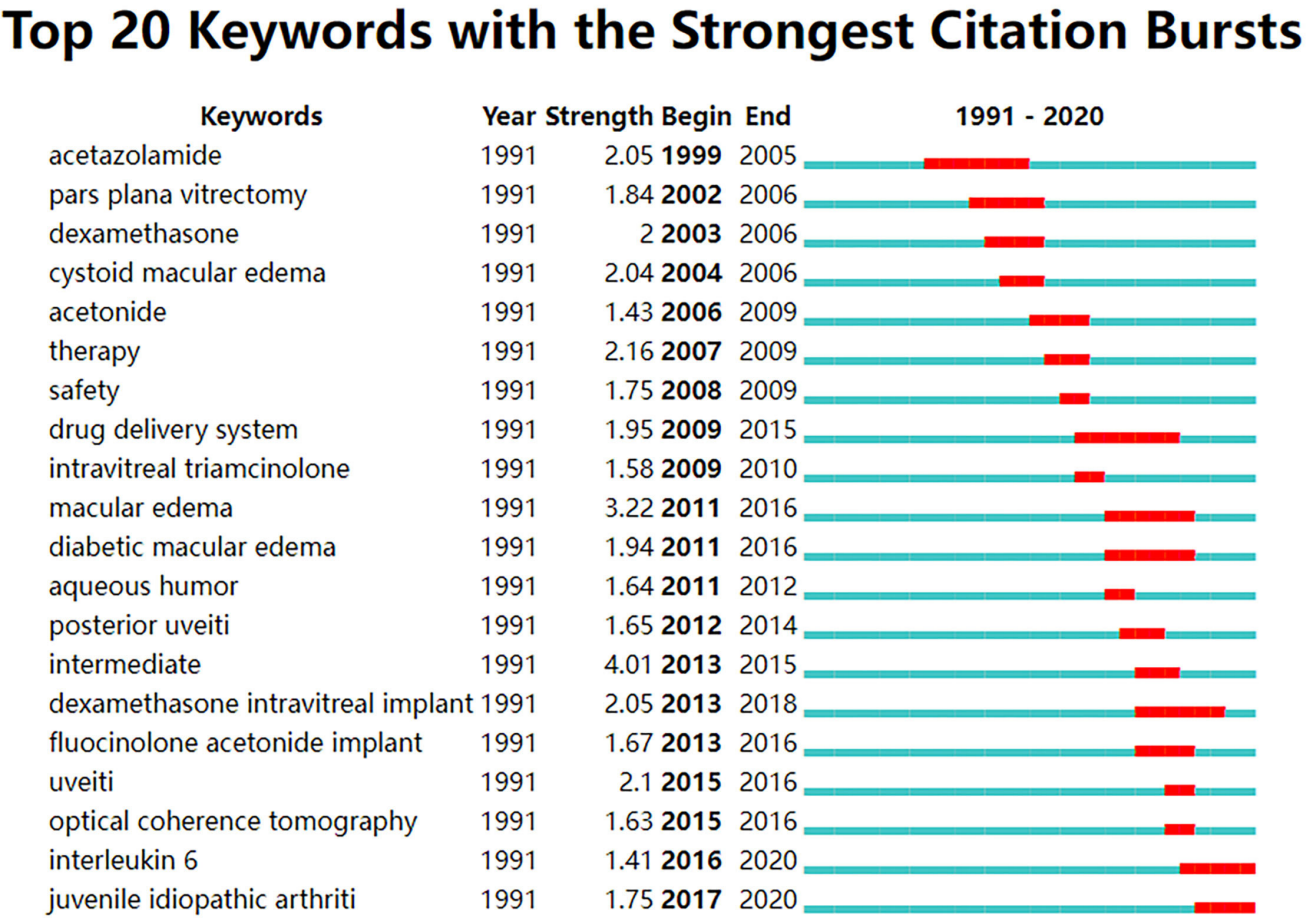
Top 20 keywords with the strongest citation bursts from the top 100 highly cited articles in the field of UME drug therapy.

Figure 4 reflects the top 20 most-cited keywords in UME drug therapy field at different stages by years. We found that the research hot spot in the field of UME drug therapy has shifted from systemic to local treatment, such as oral dexamethasone into intraocular steroid injection Meanwhile, the research hot spots of drug types is also shifting from short-acting to long-acting, for example, intraocular steroid injections from short-acting triamcinolone to long-acting fluocinolone acetonide implant.

## Discussion

In this study, we identified the top 100 cited studies in the field of UME pharmacotherapy in the WOS Core database from the beginning of the database to 2021. Bibliometrics analysis was innovatively used to evaluate the national institutions, citations, journals, keywords, and other valuable information in these literature studies, especially the hot spots and trends in drug treatment.

A medical treatment for UME was first proposed 30 years ago (21). To this day, the volume of literature published in this field is increasing year by year. Interestingly, the top institutions for publishing these highly cited articles are mainly located in London (the England) and Barcelona (Spain), but the country with the largest number of articles is the United States. This indicates that in the field of UME drug therapy, the research institutions in the England and Spain are relatively concentrated, while in the United States, they are relatively dispersed. However, this may also be related to different national conditions and research institutions. We also found that among the 100 highly cited papers, the most published papers, the most cited times, and the top three journals were all from the United States, followed by the England, the Netherlands, and Spain. All of these indicate that in the field of UME drug therapy, the United States leads in the quality of academic papers, publications, and high-impact journals. We believe this is because of three reasons as follows: (1) newly developed and produced related drugs were first marketed in the United States, such as Ozurdex and adalimumab; (2) the United States has an advantage in economic level; and (3) the US government has made policies favorable to scientific research.

Through the analysis of high-frequency keywords, it can be found that the current attention on drug treatment of UME mainly focuses on the pathway of corticosteroid administration, biological agents, and clinical verification. Despite the rapid development of immunosuppressive and biologic therapies in recent years, corticosteroids are still the main treatment for UME (22). As the field of UME drug therapy advances, the research focus of corticosteroid therapy is reflected in the way of administration. According to our research, the way steroid hormones are used is improving in a certain direction. We conclude the following two aspects: (1) in terms of drug efficacy, the focus of intraocular steroid injection has gradually shifted from short-acting drug form to long-acting sustained-release drug form, reducing the injection frequency; and (2) in terms of safety, attention is gradually shifting from systematic to local drug use, which can significantly reduce systemic side effects of patients and significantly improve safety. In particular, for patients with systemic drug intolerance or poor efficacy, intravitreal injection of corticosteroids has become a new effective choice, such as triamcinolone acetonide (TA) or DEX implant (22–24). Some studies show that the effect of intravitreal injection of TA was short-lived, but the therapeutic effect of a single injection of DEX implant lasted for more than 6 months (22, 25). Therefore, the research hot spot in the field of UME drug therapy has changed from systemic treatment to local treatment, the type of drugs has changed from short-acting to long-acting, and the clinical validation of various drugs is also the focus. Furthermore, the shift in focus from systemic treatment to local treatment may be due to the latter's less invasive nature and fewer systemic side effects. The shift from short-acting to long-acting drugs has also brought benefits to patients, such as less frequent use and less pain. At the same time, the diversification of drug types requires perfect clinical trials to ensure drug safety. Our study reveals this latest practice trend and opens up a new perspective for readers to think about.

In recent years, the research trend in the field of UME drug therapy has gradually shifted to biologic therapy, and its role has been paid more and more attention by scholars. Aleksandra Radosavljevic et al. found that biological agents such as anti-IL-6 and interferon showed positive efficacy in the majority of patients with non-infectious severe or refractory UME (26). Deuter et al. found that tozizumab can be considered for chronic UME even if immunoregulatory therapy fails (27). Thomas found that TNF-α inhibitors such as Adalimumab and infliximab may be considered as first-line therapy for Uveitis associated with Behcet disease and juvenile idiopathic arthritis (28). Diaz-Llopis et al. found that adalimumab reduces inflammatory activity and is well-tolerated in patients with refractory uveitis who are tolerant to prednisone or an immunosuppressant, and it also reduces the dose of steroids used (29). In addition, a multicenter study reported that anti-IL-6 receptor tocilizumab (TCZ) was effective in treating refractory and non-infectious uveitis CME (30). A new clinical study suggests that interferon alpha-2a is safe and well-tolerated in the treatment of refractory uveitis and macular edema, and it is an option for patients with persistent refractory UME (31). In summary, biologic therapy has many advantages, such as the rapid onset of action, rapid control of active inflammation, longer-lasting efficacy than steroid hormones, high topical safety, and fewer side effects. However, there may be cases of relapse after withdrawal, requiring multiple injections. This is one of the hot points of this study.

Although the use of intravitreal corticosteroids and biologics is becoming more common, a large number of multicenter, randomized controlled trials are still needed to evaluate the safety, efficacy, and durability of these emerging therapies due to the large individual variation in these patients. A recent multicenter steroid therapy (MUST) trial for uveitis and a follow-up study of up to 7 years showed that 94% of patients ended up with UME gone, although they were still at risk for recurrence (32). There are a wide variety of drugs to treat UME, and their risks and side effects should be considered as well as their efficacy. For example, although tocilizumab (TCZ) is effective in treating refractory UME, side effects such as nausea, viral conjunctivitis, and bullous impetigo were still observed in a few patients (27). Therefore, bibliometric analysis shows that clinical verification of drug therapy for UME is still a hot topic in future research.

## Strengths and Limitations

This article analyzes global research of UME, for the first time, by using the bibliometric analysis method. At the same time, we use the WOS database and bibliometric analysis software, both of which are objective, convincing, and more common than other databases or software. However, in our research, there are some unavoidable shortcomings. We use only one database, which may lead to the omission of some citations and documents. It is worth mentioning that due to the characteristics of the subject, the total number of articles in this field is not huge, which may be the most important reason for the limitations of this study.

## Conclusion

In this study, we used bibliometric analysis to determine the current research status and hot trends in the field of UME drug therapy. At present, the research focus of UME drug therapy is often the route of corticosteroid administration, biological agents, and clinical trials. A timely understanding of the hot trends of UME drug therapy can better help scholars to clarify the future research direction and continuously discover and solve the urgent difficulties in this field.

## Data Availability Statement

The original contributions presented in the study are included in the article/supplementary material, further inquiries can be directed to the corresponding author.

## Author Contributions

LF and SC designed this study. JK performed the search and collected data. SC, JK, and LF rechecked data. SC performed analysis. SC and JK wrote the manuscript. LF reviewed the manuscript. All authors contributed to the article and approved the submitted version.

## Funding

The work was supported by grants from the National Nature Science Foundation of China (Nos. 81870648 and 82070949).

## Conflict of Interest

The authors declare that the research was conducted in the absence of any commercial or financial relationships that could be construed as a potential conflict of interest.

## Publisher's Note

All claims expressed in this article are solely those of the authors and do not necessarily represent those of their affiliated organizations, or those of the publisher, the editors and the reviewers. Any product that may be evaluated in this article, or claim that may be made by its manufacturer, is not guaranteed or endorsed by the publisher.
